# Acute Calculous Cholecystitis Caused by *Streptococcus gallolyticus subspecies pasteurianus*: A Case Report

**DOI:** 10.3390/microorganisms10101929

**Published:** 2022-09-28

**Authors:** Tsunehiko Shigemori, Atsunori Hiasa, Yasuhiro Inoue, Satoko Oka, Taro Yasuma, Ryo Nishiwaki, Natsuko Sugimasa, Tetsuya Hamaguchi, Midori Noji, Kenji Takeuchi, Yoshiyuki Ito, Toshio Katoh, Esteban C. Gabazza, Ichiro Imoto

**Affiliations:** 1 Department of Surgery, Doshinkai Tohyama Hospital, Tsu 514-0043, Japan; 2 Department of Internal Medicine, Doshinkai Tohyama Hospital, Tsu 514-0043, Japan; 3 Department of Immunology, Mie University, Tsu 514-8507, Japan; 4 Department of Digestive Endoscopy Center, Doshinkai Tohyama Hospital, Tsu 514-0043, Japan

**Keywords:** acute calculous cholecystitis, *Streptococcus gallolyticus subspecies pasteurianus*, *Streptococcus bovis*, bilirubin calcium stone, β-glucuronidase

## Abstract

Acute cholecystitis is an infectious disease of the gallbladder caused mainly by *Escherichia coli*, *Klebsiella*, and *Enterococcus* species. *Streptococcus gallolyticus subsp. pasteurianus*, previously known as *Streptococcus bovis* biotype II/2, rarely causes endocarditis, meningitis, and septicemia, mainly in children. Biliary tract infections by *Streptococcus gallolyticus subsp. pasteurianus* are extremely rare. There have been no reports of cases in Japan. Here, we describe the first case in Japan of acute calculous cholecystitis caused by *Streptococcus gallolyticus subsp. pasteurianus* infection. A 63-year-old man was admitted to our hospital with epigastric pain and vomiting. He had moderate tenderness and a full sensation in the epigastrium. Abdominal imaging revealed multiple stones in the gallbladder. After admission, he had a high fever that did not improve with antibiotics. Percutaneous transhepatic gallbladder drainage was performed. The patient underwent open cholecystectomy. During surgery, several small stones in the gallbladder and an abscess were observed at the gallbladder base. *Streptococcus gallolyticus subsp. pasteurianus* was detected by bacterial culture of the bile juice. The gallstones were bilirubin calcium stones. The endoscopic study showed three adenomas in the colon, but the histopathological examination demonstrated no malignant cells. Although infection by this bacterium may not be rare, this is the first reported case in Japan of acute calculous cholecystitis caused by *Streptococcus gallolyticus subsp. pasteurianus* infection.

## 1. Introduction

*Streptococcus gallolyticus subsp. pasteurianus* (SGSP) belongs to the group D *Streptococcus*, formerly known as *Streptococcus bovis* type II/2 [1]. Based on biochemical differences, *S. bovis* was originally classified into type I (mannitol-fermentation-positive), type II/1 (mannitol-fermentation-negative and β-glucuronidase-negative), and type II/2 (mannitol-fermentation-negative and β-glucuronidase-positive) [1]. In 2003, Schlegel et al. proposed a new taxonomy of *S. bovis*-group bacteria based on genetic differences [2]. Although this new taxonomy for the bacterium was accepted and is currently used, many clinicians remain unfamiliar with it and therefore still call the bacterium *S. bovis*. Organisms from the *S. bovis* group have been associated with endocarditis, meningitis, sepsis, and colorectal cancers [3,4,5,6]. However, reports of biliary tract infection caused by this group of bacteria are very rare worldwide [7]. Reports of biliary tract infection by SGSP are even rarer. Although there may be many cases, herein, we present the first reported case of acute calculous cholecystitis caused by SGSP in Japan and review the literature.

## 2. Case Presentation

A 63-year-old male with a history of herpes zoster at the age of 24 and acute gastroenteritis after the ingestion of raw egg at the age of 61 was admitted to our hospital for upper abdominal pain and vomiting. The day before admission, two hours after eating a pork cutlet for dinner, he felt a burning pain in the epigastrium. Less than 24 h after the start of his symptoms, the patient consulted our institution. He continued having epigastric pain and started vomiting after admission. Physical examination revealed marked tenderness in the epigastrium and right upper quadrant but no rebound tenderness. His vital signs were within the normal range except for a body temperature of 37.3 °C. The blood analysis showed a slight elevation of leukocytes, neutrophils, C-reactive protein, triglycerides, low-density lipoprotein cholesterol, and liver enzymes (Table 1). No anemia or jaundice was observed.

**Figure 1:**
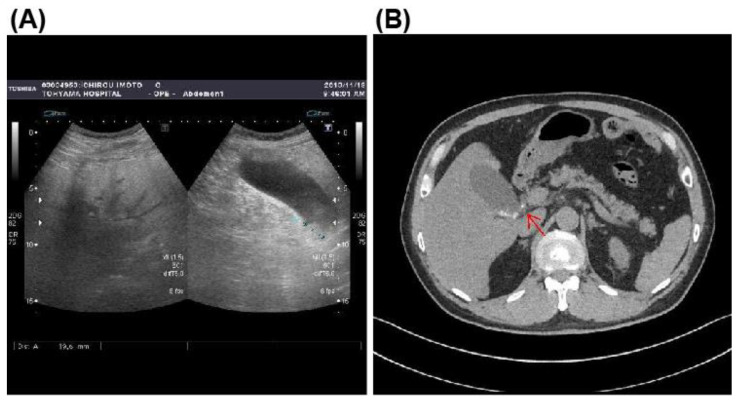
Abdominal ultrasonography and computed tomography findings. (**A**), Abdominal ultrasonography revealed moderately fatty liver and numerous small stones and sludge in the gallbladder. (**B**), Abdominal CT revealed small stones in the enlarged gallbladder with suspicion of stone impaction (arrow) in the gallbladder neck.

**Figure 2:**
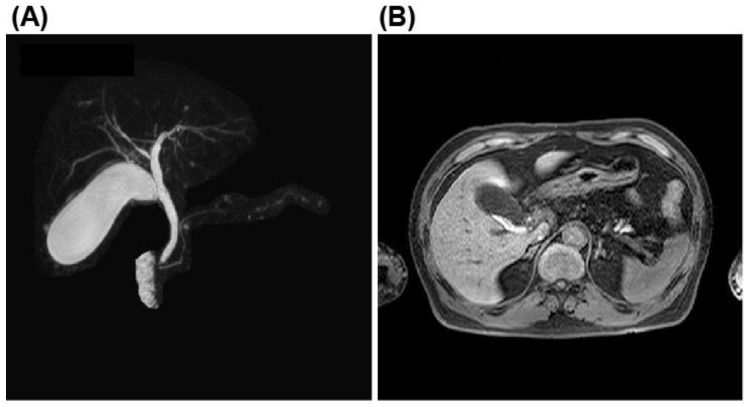
Magnetic resonance cholangiopancreatography findings. (**A**) No dilation and no stones in the common biliary duct. (**B**), Small stones and sludge in the distended gallbladder.

Abdominal ultrasonography and computed tomography (CT) findings showed a moderately fatty liver and multiple stones in an enlarged gallbladder with suspicion of stone impaction in the gallbladder neck (Figure 1A,B). However, on admission, the abdominal ultrasonography showed no typical findings of acute calculous cholecystitis, including gall bladder wall thickening, peri-cholecystic fluid, or sonographic-positive Murphy’s sign. This scarcity in typical findings was probably due to the early disease stage. Magnetic resonance cholangiopancreatography (MRCP) showed numerous stones in the gallbladder but no dilation of the common bile duct or stones in the common bile duct (Figure 2A,B). The diagnosis was acute calculous cholecystitis.

After admission, the patient received antibiotic therapy, including 1 g of ceftriaxone sodium hydrate (CTRX) twice daily. Percutaneous transhepatic gallbladder drainage (PTGBD) was performed on the same night. Sixty milliliters of turbid concentrated bile was aspirated from the PTGBD tube. The PTGBD is recommended by the Tokyo Guidelines 2018 in cases with complications or comorbidities [8]. Transhepatic needle puncture may injure hepatic blood vessels. Therefore, instead of PTGBD, percutaneous cholecystostomy is widely used for gallbladder drainage in European countries [9]. Cholecystography showed numerous small stones in the gallbladder. On the third day of hospitalization, levofloxacin hydrate 500 mg/day was added, and the fever resolved on the fourth day of hospitalization. Bile culture from the PTGBD tube revealed Gram-positive bacteria. SGSP was identified using a Vitec 2/Gram-positive (GP) identification card (Sysmex Biomérieux). The drug sensitivity test showed that the minimum inhibitory concentration of CTRX was 0.25 μg/mL. On the evening of the fifth day of hospitalization, antibiotics were switched from CTRX to 1 g of hydrochloride hydrate twice daily, which had a minimum inhibitory concentration of <0.06 μg/mL. Following these treatments, the patient’s subjective symptoms and objective findings improved.

**Figure 3:**
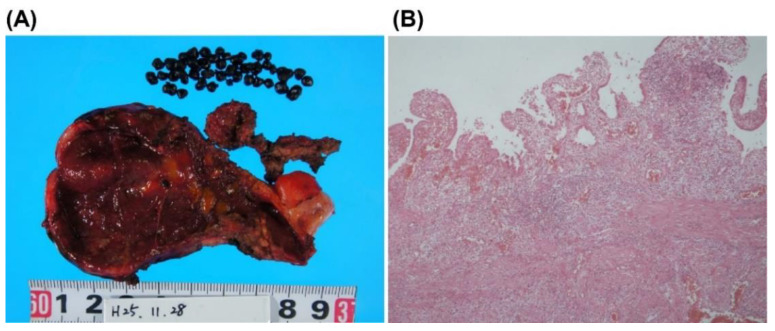
Macroscopic and microscopic findings of the gallbladder. (**A**), Macroscopic finding of the resected gallbladder. More than 30 black stones of 2–3 mm in diameter were found in the gallbladder. The base of the gallbladder was partially melted due to necrotizing cholecystitis and abscess. (**B**), Microscopic findings revealed erosion, fibrosis, and moderate inflammatory cell infiltration with neutrophils, but no malignant cells.

On the 11th day of hospitalization, the patient underwent an open cholecystectomy through a right subcostal incision. More than 30 dark brown stones 2–3 mm in diameter were found in the gallbladder, and the gallbladder base had partially melted due to necrotizing cholecystitis, forming an abscess, which was coated with a large mesh (Figure 3A). In addition, the necrotic mucosa of the gallbladder at the liver bed, which was highly inflamed, was removed as fragments. After intraoperative contrast studies confirmed the absence of residual stones, a Penrose drain was placed, and the abdomen was closed.

**Figure 4:**
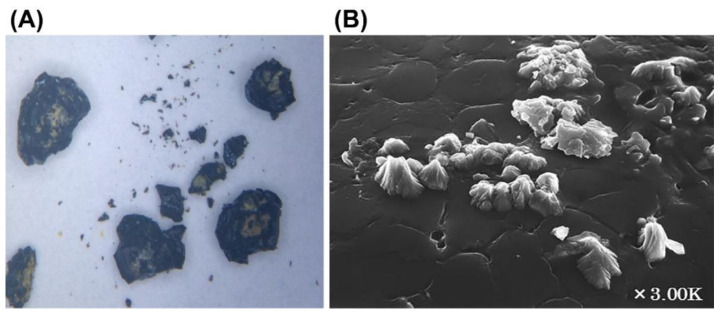
Macroscopic and scanning electron microscopic findings. (**A**), Macroscopic findings of gallstones. Black stones ranging from the size of a grain of sand to 2 to 3 mm. Brownish areas on the irregular surface of the stones. (**B**), Scanning electron microscopic findings (×3.00 K). Bacterial colonies were observed on the surface of the stone.

The histological specimens from the cholecystectomy showed erosions, fibrosis, and a moderate inflammatory cell infiltration with neutrophils, but no malignant findings (Figure 3B). Infrared spectroscopy revealed that the composition of the stones was >98% bilirubin calcium. Scanning electron microscopic analysis showed the presence of bacterial colonies on the surface of the calculus (Figure 4A). Infections caused by bacteria of the *S. bovis* group are commonly associated with colorectal cancer [6]. A colonoscopy showed the presence of three adenomas in the sigmoid colon but without malignant findings.

After removing the drain on the 12th postoperative day, the patient was discharged on the 17th postoperative day.

## 3. Discussion

In this report, we described a case of acute calculous cholecystitis caused by an SGSP (formerly *S.bovis* II/2) infection in a 63-year-old male. Microorganisms of the *S. bovis* group are commensal intestinal bacteria that can infect humans and animals, including birds [10]. This group of bacteria colonizes the intestines in 10% of healthy individuals [11]. The rate of bacteremia by this group of microorganisms is significantly correlated with the cattle density, suggesting a possible transmission of *S. bovis* from cows to people [12]. In the current case, the patient used to drink milk and eat raw eggs during childhood, although he was not engaged in raising or fattening cattle. Furthermore, *S. bovis* group members are increasingly isolated as predominant species from fermented products in Europe, Asia, and Africa [13]. Therefore, dietary habits may be involved in infection acquisition, although the route of infection is unknown.

Microorganisms that degrade gallic acid have been isolated from koalas’ intestines and named *S. gallolyticus* [1]. This bacterium was classified into three subspecies: *S. gallolyticus subs. gallolyticus*, *S. gallolyticus subs. macedonicus*, and *S. gallolyticus subs. pasteurianus* (SGSP) [1]. The latter subspecies correspond to biotype II/2 (SGSP), which was the infectious agent in the present case. Streptococci of group D, including SGSP, can grow in bile esculin agar medium containing bile acids [14]. Therefore, bacteria from the *S. bovis* group, including SGSP, can live in bile. Lee et al. evaluated 37 cases of *S. bovis* bacteremia and found that 14 (38%) had biliary tract disease, indicating that biliary tract infection was the main source of bacteremia by *S. bovis* [15]. Ruoff et al. reported that among 38 cases of bacteremia by *S. bovis*, 9 cases (23.7%) of biotype II (II/1; 7 cases, II/2; 2 cases) had a hepatobiliary source of infection [16]. Similarly, Corredoira et al. reviewed several cases of bacteremia caused by *S. bovis* and *S. salivarius* and found that *S. bovis*-biotype-II-associated bacteremia had a hepatobiliary origin in 50% of the patients [17]. In 2013, Corredoira et al. evaluated bacteria of the S. bovis group in 30 cases of cholangitis and 21 cases of cholecystitis using the VITEK 2 g-positive (GP) identification card [7]. They found 29 (57%) cases of *S. infantarius* (biotype II/1), 20 (39%) cases of SGSP (biotype II/2), and 2 (4%) cases of *S. gallolyticus subsp. gallolyticus* (biotype I). They concluded that biotype II bacteria of the *S. bovis* group are predominant in biliary tract infection.

Acute cholecystitis is a complication of cholelithiasis in more than 90% of patients [18]. Gallstones are classified according to their major components in cholesterol, pigment (calcium bilirubin stones and black stones), and rare gallstones [18]. Brown-pigment stones containing bilirubin calcium are formed in bile infected by enteric bacteria [19]. Bae et al. reported that a bile culture was positive for the following bacteria: *Escherichia coli* (25.0%), *Enterococcus* spp. (13.4%). *Klebsiella* spp. (11.1%), *Pseudomonas* spp. (11.1%), and coagulase-negative *Staphylococcus* (9.7%) [20]. Most of these bacteria have β-glucuronidase, which hydrolyzes bilirubin glucuronides and is ultimately responsible for forming calcium bilirubin gallstones [21,22]. In the current case, the gallstones contained more than 98% bilirubin calcium, and the culture was positive for SGSP, which releases β-glucuronidase that promotes the formation of gallstones.

The *S. bovis* group of bacteria has long been associated with colorectal cancer, although not all genospecies are closely related to this malignant complication. The meta-analysis reported by Boleij et al. reported four subspecies of *S. gallolyticus* and demonstrated a strong association of *S. bovis* biotype I (*S. gallolyticus sub. gallolyticus*) with the development of colorectal adenoma/cancer [3]. The association of SGSP with colon cancer was not so high [3]. We performed a colonoscopy because they are related bacteria. The endoscopic study disclosed the presence of adenoma in the sigmoid colon. However, future studies in a large population should be performed before drawing any conclusion on the association between SGSP and colorectal tumors.

## 4. Conclusions

Although infection by this bacterium may not be rare, this is the first reported case of acute calculous cholecystitis caused by SGSP infection in Japan. In addition, careful clinical follow-up is recommended due to a potential association between SGSP and the development of colorectal cancer.

## Figures and Tables

**Table 1:** Blood analysis results.

Peripheral Blood		Normal Range	Biochemistry		Normal Range
White blood cells	9800/μL	3900–9800	Total protein	7.7 g/dL	6.5–8.5
Neutrophils	81.9%	27.0–70.0	Albumin	4.7 g/dL	4.1–5.3
Eosinophils	0.5%	0.0–10.0	Total bilirubin	0.80 mg/dL	0.2–1.3
Basophils	0.3%	0.0–3.0	AST	37 IU/L	10–35
Lymphocytes	15.0%	19.0–59.0	ALT	54 IU/L	10–35
Monocytes	1.8%	0.0–12.0	Lactate dehydrogenase	174 IU/L	110–225
Red blood cells	488 × 10^4^/μL	427–570	Alkaline phosphatase	222 IU/L	110–340
Hemoglobin	15.6 g/dL	13.5–17.6	γ-GTP	30 IU/L	8–60
Hematocrit	44.5%	39.8–51.8	Cholinesterase	424 IU/L	214–466
MCV	91.3 fl	82.7–101.6	Amylase	153 IU/L	38–137
MCH	31.9 pg	28.0–34.6	Total cholesterol	233 mg/dL	150–219
MCHC	35.0%	31.6–36.6	HDL-C	69 mg/dL	40–96
Platelets	19.5 × 10^4^/μL	13.1–36.2	LDL-C	143 mg/dL	70–139
Coagulation>			Triglycerides	177 mg/dL	50–150
APTT	24.3 s	27.0–38.5	CRP	0.139 mg/dL	0.000–0.299
PT	107%	70–140	Blood urea nitrogen	9.8 mg/dL	9.0–22.0
PT-INR	0.95	0.85–1.15	Creatinine	0.64 mg/L	0.50–1.10
FDP	3.2 μg/mL	0–5.0			

MCV, mean corpuscular volume; MCH, mean corpuscular hemoglobin; MCHC, mean corpuscular hemoglobin concentration; APTT, activated partial thromboplastin time; PT, prothrombin time; PT-INR, prothrombin time–international normalized ratio; FDP, fibrin degradation products; AST, aspartate aminotransferase; ALT, alanine aminotransferase; LDH, lactate dehydrogenase; γGTP; γ-glutamyl transferase; HDL-C, high-density lipoprotein cholesterol; LDL-C, low-density lipoprotein cholesterol.

## Data Availability

All data generated or analyzed during this study are included in this published article. In addition, other material and information on this case report are available from the corresponding author on reasonable request.
